# Electron beam and thermal stabilities of MFM-300(M) metal–organic frameworks†

**DOI:** 10.1039/d4ta03302g

**Published:** 2024-07-01

**Authors:** Eu-Pin Tien, Guanhai Cao, Yinlin Chen, Nick Clark, Evan Tillotson, Duc-The Ngo, Joseph H. Carter, Stephen P. Thompson, Chiu C. Tang, Christopher S. Allen, Sihai Yang, Martin Schröder, Sarah J. Haigh

**Affiliations:** a Department of Materials, The University of Manchester Oxford Road Manchester M13 9PL UK sarah.haigh@manchester.ac.uk; b Department of Chemistry, The University of Manchester Oxford Road Manchester M13 9PL UK; c Diamond Light Source Ltd Diamond House, Harwell Science and Innovation Campus Didcot Oxfordshire OX11 0DE UK; d Department of Materials, University of Oxford Oxford OX1 3PH UK; e Electron Physical Science Imaging Centre, Diamond Light Source Ltd Didcot Oxfordshire OX11 0DE UK

## Abstract

This work reports the thermal and electron beam stabilities of a series of isostructural metal–organic frameworks (MOFs) of type MFM-300(M) (M = Al, Ga, In, Cr). MFM-300(Cr) was most stable under the electron beam, having an unusually high critical electron fluence of 1111 e^−^ Å^−2^ while the Group 13 element MOFs were found to be less stable. Within Group 13, MFM-300(Al) had the highest critical electron fluence of 330 e^−^ Å^−2^, compared to 189 e^−^ Å^−2^ and 147 e^−^ Å^−2^ for the Ga and In MOFs, respectively. For all four MOFs, electron beam-induced structural degradation was independent of crystal size and was highly anisotropic, although both the length and width of the channels decreased during electron beam irradiation. Notably, MFM-300(Cr) was found to retain crystallinity while shrinking up to 10%. Thermal stability was studied using *in situ* synchrotron X-ray diffraction at elevated temperature, which revealed critical temperatures for crystal degradation to be 605, 570, 490 and 480 °C for Al, Cr, Ga, and In, respectively. The pore channel diameters contracted by ≈0.5% on desorption of solvent species, but thermal degradation at higher temperatures was isotropic. The observed electron stabilities were found to scale with the relative inertness of the cations and correlate well to the measured lifetime of the materials when used as photocatalysts.

## Abstract 2

This work reports the thermal and electron beam stabilities of a series of isostructural metal–organic frameworks (MOFs) of type MFM-300(M) (M = Al, Ga, In, Cr).

## Graphical abstract

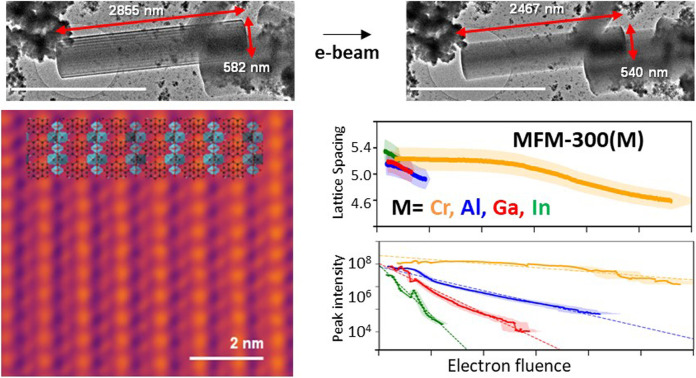

## Introduction

Metal–organic frameworks (MOFs) are a class of nanoporous crystalline materials typically composed of metal cations connected by organic linkers in a three-dimensional network.^1^ These crystals have controllable pore sizes of the order of 1–9 nm,^2^ making them highly promising for applications in gas adsorption and separation for sustainable energy applications,^3^ heterogeneous catalysis,^4^ and photocatalysis.^5^ Control of composition and synthesis conditions allows the MOF's pore geometries to be precisely modified to optimise performance for a particular reaction,^6,7^ while defect engineering provides the ability to further tune their chemical properties for various applications in adsorption,^8,9^ catalysis,^10,11^ and photocatalysis.^12^

Despite the importance of defects in controlling the material's performance, the characterisation of local defects within MOF crystals remains challenging. X-ray diffraction (XRD) techniques provide average structural data for MOFs of sufficiently large crystal size,^13,14^ with further structural understanding of pore geometries being gained *via* gas adsorption isotherms.^13,14^ Chemical information on defects can be obtained using bulk spectroscopic analysis methods such as infrared (IR),^15,16^ ultraviolet-visible light (UV-vis),^13^ and nuclear magnetic resonance (NMR) spectroscopy,^17^ whereas thermogravimetric analysis (TGA) can provide information about thermal stability under various environmental conditions.^18^ However, these techniques all provide information averaged across a bulk volume of the sample, making them unsuitable for probing local structural information on crystal defects and how the crystal structure degrades when subject to harsh operating conditions.

Transmission electron microscopy (TEM) is uniquely able to provide atomic-scale visualisation of local pore and surface structures and localised defects.^19–22^ Electron diffraction in the TEM provides local crystallographic structural information,^23,24^ and both energy-dispersive X-ray spectroscopy (EDXS)^25^ and electron energy-loss spectroscopy (EELS)^26^ can provide spatially resolved chemical information. Despite this potential, however, TEM characterisation is not widely applied to MOFs, likely due to their relatively high susceptibility to damage from the high-energy electron beam during the TEM experiment.^27^

Degradation of the crystal structure of MOFs has been found to occur at incident electron fluences similar to those needed for imaging of proteins, which is on the order of 10 e^−^ Å^−2^,^28,29^ whereas conventional high-resolution TEM (HR-TEM) imaging typically requires fluences on the order of 10^4^ e^−^ Å^−2^.^30^ However, recent developments have improved the efficiency of electron detectors and have made the imaging of MOFs more accessible.^31,32^ These include direct electron detector (DED) cameras,^33,34^ and segmented detectors for integrated differential phase contrast scanning transmission electron microscopy (iDPC-STEM).^35,36^ Despite these advances, only one study has quantitatively compared the effects of different benzene-based linkers on the electron beam degradation of Cu-based MOFs.^37^ Deeper understanding of the electron beam-induced degradation behaviour remains an essential step to ensure that the high-resolution TEM data obtained is correctly interpreted and does not contain damage artefacts.

We report herein the use of TEM electron diffraction and imaging to quantify the electron beam-induced degradation pathway for the MFM-300 series of MOFs. We demonstrate that electron beam-induced structural damage occurs anisotropically and discuss how this relates to the resultant changes in the pore structure. We study crystal size effects and find that size is not a factor in determining the stability of the materials. We also quantitively study thermally-induced changes to the crystal structure and find that although this is in good agreement with the electron stability trends for different MOF cations within the same group in the periodic table, the inertness of the cation when interacting with solvent ions is a better prediction of the stability of the MOF under the electron beam.

## Experimental methods

### Synthesis of MFM-300(M) MOFs

MFM-300(Al),^14,16^ MFM-300(Ga),^38^ MFM-300(In)^15^ and MFM-300(Cr)^39^ were synthesised *via* solvothermal reaction according to previously reported methods. Further details of the synthetic methods can be found in ESI 1.†

### Transmission electron microscope imaging and electron diffraction series acquisition

TEM samples were prepared by using glass pipettes to drop cast suspensions of the samples onto copper TEM grids covered with a holey carbon support film. A series of electron diffraction patterns of the crystals was acquired with a [110] viewing direction using an FEI Tecnai G2 20 TEM operated at 200 kV and equipped with a LaB_6_ thermionic filament and Gatan Orius CCD camera. A 40 µm diameter selected area aperture was used for acquisition of the electron diffraction patterns. Measurement of the accumulated electron fluence was initiated as soon as the crystal was exposed to the electron beam. Aligning the MOF crystals to a common zone axis was necessary to ensure consistent data collection and cross-comparison of the resulting diffraction intensities. This required the diffraction pattern to be visible on the CCD camera, necessitating a minimum electron flux of 0.10 ± 0.01 e^−^ Å^−2^ s^−1^. This same electron flux was used for acquisition of all the electron diffraction series presented in this work. Each diffraction pattern in the series was captured using an exposure time of 30 s (total fluence of 3.0 ± 0.3 e^−^ Å^−2^ per pattern) at 1 min intervals. Crystal alignment took 2–12 min (12–72 e^−^ Å^−2^), so images at or near *t* = 0 s are missing from all datasets. The whole crystal was uniformly illuminated throughout data acquisition, which continued until no crystal structure information was visible in the diffraction pattern above the signal-to-noise ratio (SNR) of the CCD camera (1–4 h depending on the MOF sample). See ESI 2† for more information about how the TEM was calibrated to estimate the electron fluence.

In order to quantify the decay in electron diffraction intensity, automated peak identification and intensity analysis was carried out in Python 3.10 with the aid of functions and modules from various signal and data analysis packages.^40–45^ The intensities of the individual peaks, accounting for the local background intensity due to scattering from amorphous material, were measured as a function of the accumulated electron fluence along with their changes in position. A beam stop was used to prevent the high intensity of the directly transmitted beam from damaging the CCD camera and where this obstructed individual diffraction peaks, only those peaks present in all datasets were used in the analysis. See ESI 3† for full discussion of diffraction pattern analysis methods.

Bright-field scanning transmission electron microscope (BF-STEM) imaging was used to reveal the lattice structure. These images were acquired on a JEOL GrandARM 300, operated at an accelerating voltage of 300 kV. Crystals of interest were aligned in TEM imaging mode before switching to STEM mode for the image acquisition. The total electron flux for alignment was estimated as <100 e^−^ Å^−2^, and individual images had a total flux of ≈60 e^−^ Å^−2^ (512 × 512 pixels, 1.9 pA probe current, 3 µs pixel dwell time). These were then denoised using a patch-based principal component analysis (PCA) approach described previously.^46^

### Thermal analysis

Thermogravimetric analysis (TGA) for each material was performed by heating with a TA SDT 650 thermal analyser from room temperature to 800 °C under N_2_ at a heating rate of 240 °C h^−1^. A Savitsky–Golay filter was applied to the raw data for mass loss to minimise the effects of noise when calculating the rate of mass loss of the crystals as a function of temperature. Additionally, variable temperature powder X-ray diffraction (VT-PXRD) patterns were collected for crystals of MFM-300(M) at the I11 beamline, Diamond Light Source, using a heating rate of 300 °C h^−1^ from 25 °C to 800 °C. The diffraction patterns were indexed using unit cell data of vacant variants of the MFM-300 (see ESI 7† for links to the relevant files).

## Results and discussion

### Quantifying electron beam-induced crystal degradation

**Fig. 1:**
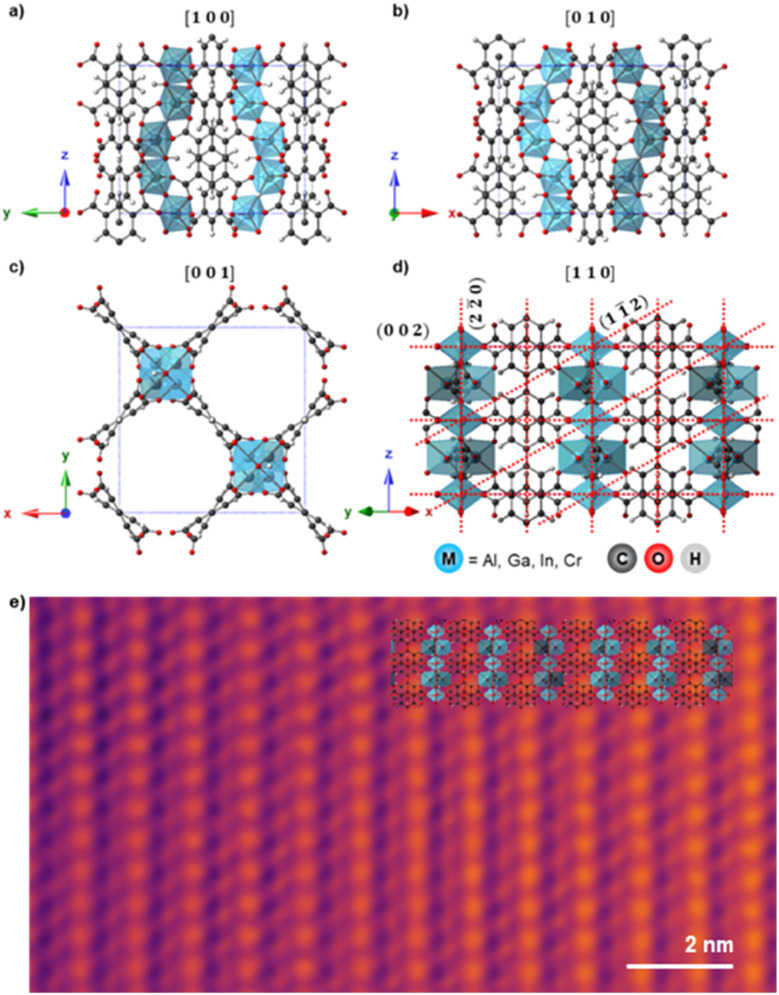
a)–(d) Schematic atomic models for MFM-300(M) as viewed along the (a) [100], (b) [010], (c) [001], and (d) [110] directions. The (002), (11̄2), and (22̄0) planes are indicated by red dashed lines in (d). The view in (c) is along the main pore channel direction and the long axis of the crystals, while views in (a)–(c) are perpendicular to this direction. Crystallographic models are presented for MFM-300(Al) but other MOFs studied are isostructural.^16^ (e) Denoised bright-field STEM image of MFM-300(Cr) aligned along [110], with the structural model overlaid. Total fluence used for the image was 53.8 e Å^−2^.

**Fig. 2:**
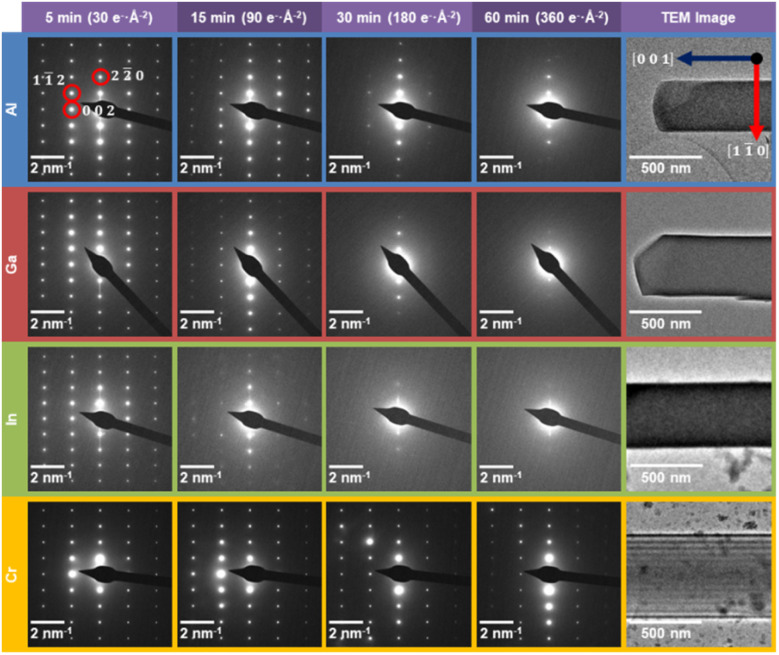
Electron beam induced evolution of the selected area electron diffraction patterns as a function of electron beam irradiation for MFM-300(M). Electron diffraction patterns of MFM-300(Al) (blue, first row), MFM-300(Ga) (red, second row), MFM-300(In) (green, third row), and MFM-300(Cr) (yellow, fourth row) over time (5, 15, 30, and 60 min) under constant exposure to an electron beam of incident flux 0.1 e^−^ Å^−2^ s^−1^. The crystals from which the diffraction patterns were acquired are displayed in the TEM images in the right-hand column, with the in-plane lattice vectors illustrated on the image for MFM-300(Al). All crystals are viewed down the [110] zone axis. The corresponding acquisition time and total electron fluence for the diffraction patterns is given above each column. The 22̄0, 11̄2, and 002 reflections are highlighted by the red circles on the top left, 5 min MFM-300(Al) diffraction pattern. The diffraction patterns and TEM images have been rotated such that they are all viewed in the same orientation.

Fig. 1a–d shows the unit cell structure of MFM-300(M) viewed down the [100], [010], [001], and [110] directions respectively, as previously reported from XRD analysis.^14,15,38,39^ MFM-300(M) crystallises in the tetragonal space group *I*4_1_22, with the metal cations arranged in spiralling chains running parallel to the pore directions along the [001] direction (Fig. 1c). These cations are connected *via* hydroxyl bridges and biphenyl-3,3′,5,5′-tetracarboxylate linkers, forming the (nn̄0) planes when viewed along the [110] direction (Fig. 1d). The octahedral coordination environment of the cations is shown by the blue polyhedra in Fig. 1. The crystal is highly anisotropic, containing one-dimensional channels parallel to the [001] lattice direction, with these channels having an almost square cross section of width ≈6.5–8.1 Å, depending on the cation M.^47^ The location of the cationic chains which form the channels and the tetracarboxylate linkers are visible in the bright-field scanning transmission electron microscope (BF-STEM) image in Fig. 1e, demonstrating the potential of TEM/STEM to visualise local atomic structures in these materials. All the MFM-300(M) materials were rod-shaped crystals of 200–800 nm width and 1–3 µm long, as confirmed by the TEM images (Fig. 2, right).

Minor peak splitting was observed along the nn̄0 reciprocal lattice directions in the diffraction pattern for MFM-300(Cr) (Fig. S4†). Measurements of the lattice spacing corresponding to the two sets of peaks reveals that they correspond to (110) spacings of 10.60 Å and 10.75 Å (Table S2†). The former matches those calculated from reported structural data,^39^ whereas the latter could suggest the expansion in diameter of the channels in MFM-300(Cr). This was recently reported for other isostructural members of this series of materials.^48^ No evidence of such peak splitting was observed for the MOFs with Group 13 cations.

The selected area electron diffraction patterns (Fig. 2) reveal the structural changes that occur for each of the MFM-300(M) materials after 5, 15, 30, and 60 min of electron exposure (accumulated electron fluences of 30, 90, 180 and 360 e^−^ Å^−2^ respectively). In general, smaller lattice spacings (high spatial frequency data furthest from the centre of the diffraction pattern) are expected to reduce in intensity more quickly than larger spacings, since a fixed atomic displacement will have a greater disruptive effect for smaller lattice separations.^49^ However, the diffraction patterns in Fig. 2 reveal a clear anisotropy in the degradation behaviour for all MOFs studied. The (nn̄0)-type lattice planes are the most robust features across all MFM-300(M) diffraction patterns, corresponding to the lattice planes parallel to the long axis of the crystals.

**Fig. 3:**
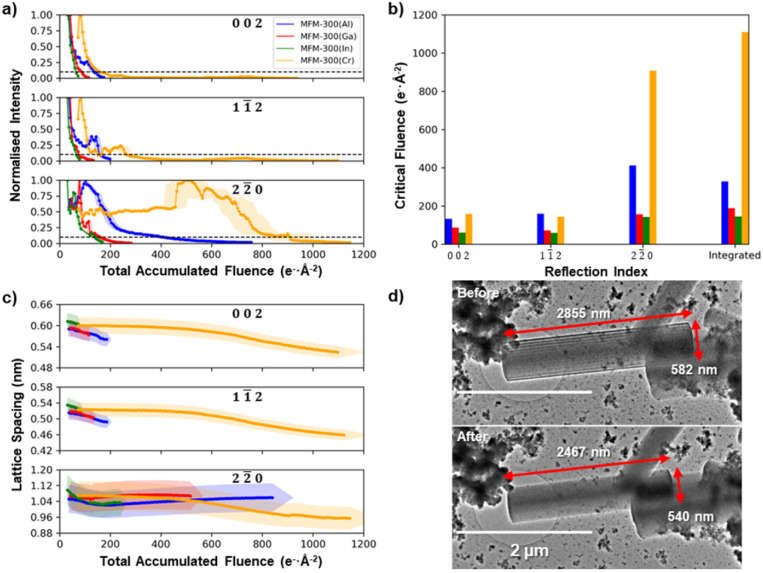
Anisotropic degradation of the MFM-300(M). (a) Decay of the electron diffraction spot intensity of the 002, 11̄2, and 22̄0 reflections for MFM-300(M) as a function of the total accumulated electron fluence. (b) The critical electron fluences at which the diffraction intensities for the 002, 11̄2, and 22̄0 reflections as well as the integrated intensity drops to 10% of their maximum intensity for MFM-300(M) crystals. (c) The evolution of the 002, 11̄2, and 22̄0 lattice spacings as a function of the total accumulated electron fluence. (d) TEM images before and after electron irradiation illustrating the physical shrinkage of a crystal of MFM-300(Cr) with dimensions indicated on the images. Shading in (a) and (c) represents error bars on the measurements.

Fig. 3a illustrates the decay of the intensities for the 002, 11̄2, and 22̄0 reflections as a function of the total accumulated electron fluence for each of the four MFM-300(M) MOF crystals. Fig. 3b gives the critical electron fluence for each reflection, *σ*_c_, defined here as the fluence at which the measured intensity degrades to 10% of its maximum intensity. All four MFM-300(M) crystals show critical fluences that are similar for both 002 and 11̄2 reflections (*σ*_c_(002) ≈ *σ*_c_(11̄2)), while the 22̄0 reflections all have more than double the critical fluence for that material (*σ*_c_(22̄0) > 2.2 *σ*_c_(002) ≈ 2.2 *σ*_c_(11̄2)), irrespective of choice of cation. For example, in MFM-300(Al), the 002 and 11̄2 reflections decay with a critical fluence of 134 and 160 e^−^ Å^−2^ respectively, while the 22̄0 reflection has a much higher critical electron fluence of 413 e^−^ Å^−2^. Some unexpected increases in diffraction intensity are observed superimposed on the decay during these experiments (particularly notable at a fluence of 450 e^−^ Å^−2^ for the 22̄0 reflections in MFM-300(Cr)). However, as the fluence that these occur at is not reproducible, they are likely a result of crystal tilt during the degradation process.

Analysis of the diffraction pattern degradation reveals a change in the unit cell dimensions of MFM-300(M) as a function of electron beam irradiation fluence for all cations. Fig. 3c shows how the lattice spacings corresponding to the 002, 11̄2, and 11̄0 reflections change as the crystal is irradiated. The (002) lattice spacings for all MFM-300(M) materials showed continuous lattice shrinkage during electron beam irradiation, with the largest contraction of ≈13% seen for the MFM-300(Cr), with smaller shrinkages of ≈5%, ≈3% and ≈1% for M = Al, Ga, and In, respectively. MFM-300(Cr) also shows a continuous lattice shrinkage of 10% for the perpendicular 22̄0 lattice reflections while the Group 13 element MOFs show no change for MFM-300(Ga) and an initial shrinkage followed by a slight recovery for MFM-300(Al) and MFM-300(In). This shrinkage is also translated into a macroscopic change in crystal shape after electron beam irradiation. Fig. 3d compares TEM images of the MFM-300(Cr) crystal before and after diffraction series acquisition, and reveals a decrease in the long axis of 13.6% and a decrease in width of 7.2%. These illustrate shrinkages of the (002) and (22̄0) lattice spacings, respectively, and are in good agreement with the lattice spacing shrinkage observed in the X-ray diffraction patterns.

Despite the lattice shrinkage, the MOF crystallites retain their approximate shape even at electron fluences several orders of magnitude larger than that required to remove all the diffraction spots from the SAED pattern. Bubble formation (*i.e.* trapped gas evolution due to MOF decomposition) was not observed during electron beam irradiation. Together with the strong diffuse scattering present in the diffraction data beyond the critical electron fluences, this suggests that the porous structure amorphizes without significant changes in composition.

The observed anisotropic crystal deformation can be related to the crystal structure of MFM-300(M). The columns of metal cations (Al, Ga, In, or Cr) run parallel to the [001] directions, giving rise to the (22̄0) planes when viewed along [110], as illustrated schematically in Fig. 1d. These cation-containing (22̄0) planes are found to be the most structurally stable element of the MOF during electron irradiation, such that these lattice planes, and the one-dimensional channels that they delineate, persist even when all other long-range order in the material has degraded. This is likely related to the columns of metal cations being less susceptible to radiolytic electron beam-induced degradation compared to the organic linkers.

**Fig. 4:**
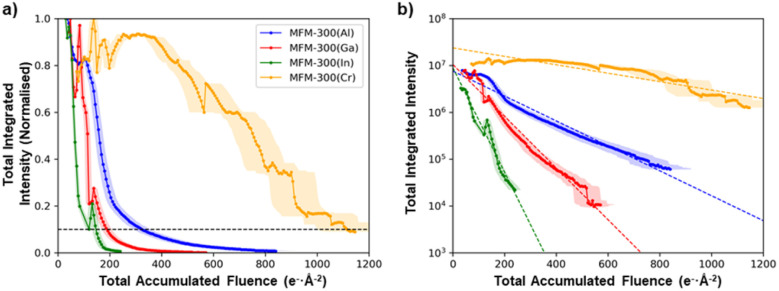
Evolution of the total integrated intensities of the MFM-300(M) materials as a function of the total applied electron fluence, (a) normalised to the maximum intensity and (b) plotted on a log scale. The anomalous oscillations in intensity observed as the crystals degrade are likely the result of electron beam damage-induced tilting of the crystal, which slightly changes the diffraction conditions.

Although all the MFM-300(M) materials showed qualitatively the same anisotropic degradation behaviour, with (nn̄0)-type lattice planes being the most structurally stable, there were large differences in their critical fluence to complete amorphization (Fig. 3b). To improve the accuracy of quantitative comparison for the different cation chemistries, the summed diffraction intensity in the reflections was measured as a function of the total electron fluence (Fig. 4a). MFM-300(Al) was found to have the highest critical fluence of the three metal cations from Group 13, with a summed critical fluence of 330 e^−^ Å^−2^. The stability of the MOFs decreased with increasing cation size, with MFM-300(Ga) and MFM-300(In) having summed critical fluences (*σ*_c_(sum)) of 189 e^−^ Å^−2^ and 147 e^−^ Å^−2^, respectively. Interestingly, the substitution of Cr, a Group 6 element, made the MOF significantly more stable, with MFM-300(Cr) having the highest critical fluence of 1111 e^−^ Å^−2^.

**Fig. 5:**
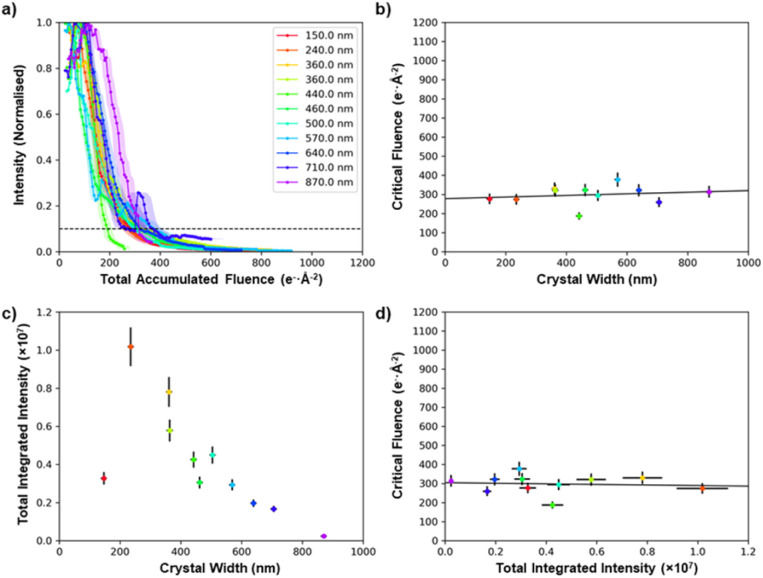
Comparison of the stability of MFM-300(Al) as a function of crystal size. (a) The normalised decay of the total diffraction intensity as a function of the total electron fluence. (b) The critical fluence of each crystal as a function of its width/thickness. (c) Comparison of how the initial intensity as measured in the diffraction spots varies as a function of its width/thickness. (d) Critical fluence as a function of initial integrated intensity. All crystals were imaged with an electron flux of 0.1 e^−^ Å^−2^ s^−1^.

Specimen thickness could influence the stability of the crystal under the electron beam,^50,51^ but in general it is not straightforward to estimate the crystal thickness from a projected TEM image. However, cross-sectional SEM imaging of individual MFM-300(M) crystals (see ESI 5†) showed that they all have a square cross-sectional geometry, and this allows the projected thickness to be calculated from the crystal width when viewed along a known crystal axis. This makes it possible to quantify the effect of crystal thickness on critical fluence. Fig. 5 shows the comparison of 11 MFM-300(Al) crystals of different thicknesses (100–900 nm). Thickness was found to be a weak effect, with thicker crystals being only slightly more stable; the mean trend line corresponds to an increase in critical fluence of only 4.2 e^−^ Å^−2^ per 100 nm increase in thickness (equivalent to ≈1% of the integrated critical fluence), while the difference in critical fluence for different crystals of similar thicknesses (400–500 nm) was >120 e^−^ Å^−2^ (equivalent to ≈30% of the integrated critical fluence). This suggests that factors other than thickness have a greater impact in determining the degradation behaviour of individual crystals. This variability is potentially related to the presence of adsorbed species in the pores and on the surface, which has been shown to decrease electron beam stability of zeolitic frameworks,^52–54^ although to our knowledge this effect has not been studied for MOFs. It should be noted that despite crystal size having only a small effect on the stability of the material, our comparison of MOF degradation behaviour shown in Fig. 2–4 used crystals which all had similar thicknesses of 400–500 nm.

Comparing the initial summed intensities of the 11 MFM-300(Al) crystals as a function of thickness reveals that in the range 200–900 nm, thinner crystals have a higher integrated diffraction intensity (Fig. 5c). The direct beam is not included in the integrated diffraction intensity, so this is a measure of the change in intensity of the diffraction spots due to inelastic scattering processes, and hence provides a guide to the increasing proportion of low-energy electrons generated for thicker samples. Low-energy electrons are more likely to result in radiolytic damage, while the higher energy electrons predominantly cause knock-on damage.^55,56^ As the critical fluence is only weakly dependent on thickness, this suggests that the damage mechanism here is a combination of both radiolytic and knock-on damage processes. The thinnest crystal we were able to find in this study (150 nm) has a comparatively low intensity, which is not unexpected as the mean free path of a 200 kV electron beam in MFM-300(Al) is certainly greater than 100 nm. This indicates that for such thin crystals a high fraction of the electrons will be unscattered and remain in the directly transmitted beam, which is not measured in the data in Fig. 5c.

### Quantifying thermal degradation behaviour

**Fig. 6:**
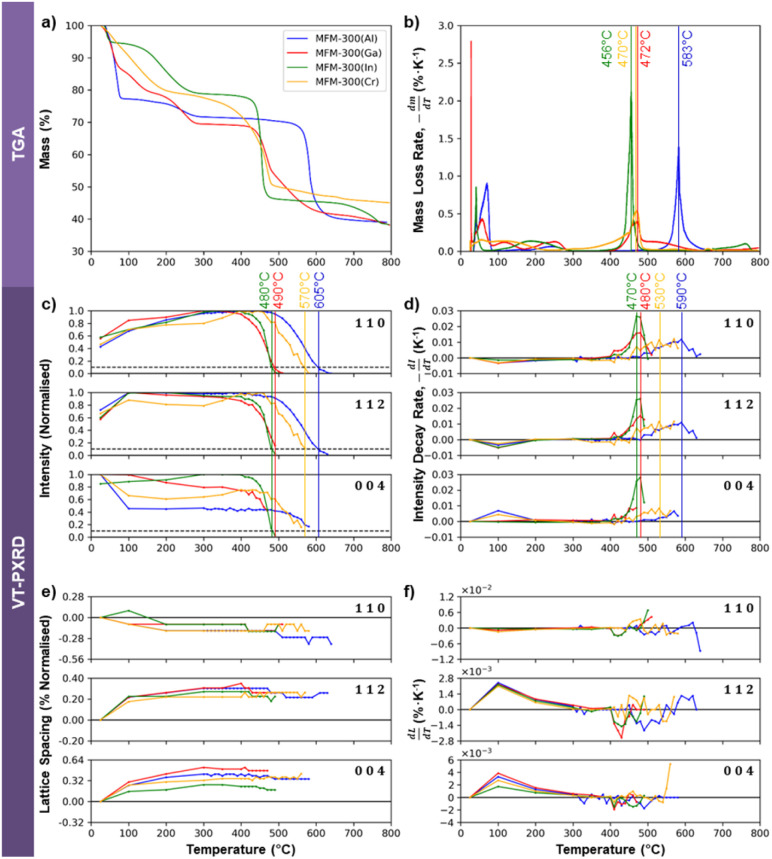
Analysis of the thermally-induced degradation behaviour of MFM-300(M) using TGA (a and b) and VT-PXRD (c–f). (a) Mass loss of the sample on heating. (b) Mass loss rate of (a) as a function of temperature, with the temperatures at which peak mass loss rate occurred being taken to be the degradation temperature and labelled. (c) Evolution of normalised peak intensities with temperature, with the 10% critical threshold line shown. (d) Intensity decay rate of the peaks in (c), with the temperatures at which the peak decay rate was observed being labelled. (e) The evolution of the 110, 112, and 004 lattice spacings with temperature, normalised as a percentage with respect to the initial value. (f) The rate of change of the normalised lattice spacings, from which the linear thermal expansion coefficients of the MOFs along the specified directions can be determined.

Electron beam irradiation can induce local heating, although the degree of heating depends on the sample size, shape, and support material.^57^ Thermogravimetric analysis (TGA) was therefore used as an independent means to test whether the electron beam stability of the four MOFs correlates with their thermal stability. The TGA data in Fig. 6a and b show some mass loss below 300 °C, which can be attributed to the desorption of solvent molecules from within the framework structures. Beyond 300 °C, mass loss is understood to be due to structural decomposition. MFM-300(Al) is the most thermally stable of the Group 13 MOFs, with decomposition occurring at 583 °C. MFM-300(Ga) is the next most stable, breaking down at 472 °C, while MFM-300(In) degrades at 456 °C. The relative thermal stabilities of these three crystals thus correlates well to their relative electron beam stabilities, with MFM-300(Al) being the most stable and MFM-300(In) being the least stable. MFM-300(Cr), however, decomposes thermally at 470 °C, giving it a similar thermal stability to MFM-300(Ga). This contrasts with its exceptionally high electron beam stability of more than 2 times that of MFM-300(Al) and more than 4 times that of MFM-300(Ga) (Fig. 3b).

TGA analysis does not provide any direct crystallographic information about these materials, so variable temperature powder X-ray diffraction (VT-PXRD) was performed to better understand the effect of temperature on the crystal structure of the MOFs. Plotting the normalised VT-PXRD peak intensities as a function of temperature allows determination of the “critical temperature” for crystal structure degradation for each MOF (defined when the intensity of the VT-PXRD peaks falls to 10% of the maximum value). Fig. 6a shows the critical degradation temperatures for MFM-300(M) to be 605, 570, 490 and 480 °C for Al, Cr, Ga, and In variants respectively, revealing that MFM-300(Al) crystallites are the most thermally stable, while the Ga and In analogues degrade at lower temperatures. This is in good agreement with their relative stabilities shown by the mass loss rates observed by TGA (Fig. 6b). However, MFM-300(Cr) is more stable than would be expected from the TGA data, suggesting it may be sensitive to the rate of heating (the TGA used a slightly lower ramp rate). An alternative approach that is more suitably correlated to the TGA measurements of mass loss rate is obtained by evaluating the first order differential of the intensity change, as shown in Fig. 6d. Using this method, we can see that the critical temperature for degradation of the Al, Ga, and In MOFs, defined here as the temperature at which the decay rate reaches its peak, are generally in good agreement (within 14 °C) of the TGA data. MFM-300(Cr) is again the outlier when this analysis approach is used for the VT-PXRD, showing higher stability than expected from the TGA.

The thermal stability of a series of M-BDC MOFs (BDC^2−^ = benzene-1,4-dicarboxylate; M = Al, Cr and V) has previously been reported to correlate well to the enthalpy of formation of their respective metal oxides,^58^ with increasingly exothermic enthalpies being indicative of increasing MOF thermal stability. We observe the same trend for the four MOFs studied here (Al_2_O_3_ = −1675.7 kJ mol^−1^; Cr_2_O_3_ = −1139.7 kJ mol^−1^; Ga_2_O_3_ = −1089.1 kJ mol^−1^; In_2_O_3_ = −925.8 kJ mol^−1^),^59^ suggesting the thermal degradation tendency we observe may also be explained by the strength of the metal–oxygen bond in common oxides.

Comparing the relative intensities of the 110, 112, and 004 PXRD peaks as a function of temperature for the four MFM-300(M) crystals can reveal details of their structural stability. Fig. 6c and d show a decrease in the intensity of the 004 reflection and an increase in the 110 and 112 at ≈100 °C. This is particularly notable in MFM-300(Al) and MFM-300(Ga), and is likely associated with the loss of adsorbed solvents. As the (110) planes are the walls of the one-dimensional channels, this is consistent with the homogeneity of the channels being increased by removing these solvents. The diffraction angles also change slightly around 100 °C as shown in Fig. 6e, which reveals a 0.1–0.2% thermally-induced shrinkage of the 110 lattice spacings and a 0.2–0.5% increase of the 004 spacing between room temperature and 300 °C. This demonstrates a reduction in diameter of the one-dimensional channels (*a*- and *b*-axes) accompanied by an expansion along the channel length (*c*-axis) with the overall volume change of the crystal found to be small (less than 0.05%, reaching a maximum at 100 °C, Fig. S9†). This evidence of a slight reduction in the width of the one-dimensional pores around 100 °C suggests the presence of adsorbed solvents at room temperature acting to slightly increase the internal diameter of the MOF channels.

Above 300 °C all solvents are expected to be evacuated and no further changes are discerned in the observed lattice spacings until onset of thermal degradation (Fig. 6c and d). In this regime, all peaks increase and decay together, demonstrating that the crystallinity degrades isotropically in response to elevated temperature, in direct contrast to the observed anisotropic electron beam-induced degradation behaviour.

### Factors affecting crystal degradation

Our results confirm that, overall, the thermal degradation and electron beam degradation behaviours for the Group 13 (Al, Ga, and In) MOFs are in good agreement, with MFM-300(Al) being the most stable and MFM-300(In) being the least. This order of relative stability is correlated to the relative stabilities of the corresponding oxide phases, which has been reported to predict the thermal stabilities of MOFs.^59^ However, the transition metal-containing MOF, MFM-300(Cr), shows higher electron beam stability than would be expected from its thermal degradation behaviour, and it is interesting to consider what other factors might be contributing to this effect.

MOF chemical stability has been reported to correlate to the inertness of the metal cation when interacting with a solvent, which can be defined as the mean lifetime, *τ*, of a particular solvent molecule in the first coordination shell of a given metal ion complex, with higher values corresponding to greater inertness.^60^ The inverse of inertness, and thus of *τ*, is lability, which is measured by the solvent exchange rate, *k*, defined as the mean number of solvent molecules exchanged per second, with water being the standard solvent molecule used for these comparisons.^60^ Considering the cations used in our work, Cr^3+^ has the lowest reported water exchange rate of the four metal cations, followed by Al^3+^, Ga^3+^, and In^3+^,^60–62^ which agrees with the electron beam stabilities of the MOFs measured in this study. Al^3+^, Ga^3+^, and In^3+^ all have fully populated inner electron shells, so the decrease in their cation inertness can be attributed both to their increased ionic radii,^60,61^ and to the increasing inert s-pair effect going down the group, which causes the +3 oxidation state to become less stable in the heavier group members.^63^ In the case of electron beam stability, lability can be indicative of the ease with which the metal cations react with radiolytic species generated by the electron beam, with greater cation lability indicating greater susceptibility to electron beam-induced structural degradation. This would suggest that isostructural MFM-300(M) MOFs with M = Rh^3+^ or Ir^3+^ could potentially be more resistant to electron beam irradiation due to their low water exchange rates, while MFM-300(Sc) would be less stable due to Sc^3+^ having a similarly high lability to In^3+^,^61^ although both hypotheses would need to be experimentally verified.

Other factors that might affect the electron beam stability of the MOFs are thermal and electrical conductivities. The development of thermally^64^ and electrically^65,66^ conductive MOFs are active areas of research, as these would broaden the application of their highly tuneable gas adsorption behaviours for both electro- and photocatalysis. While the thermal conductivities of the MFM-300(M) are unknown, MOFs generally have low conductivities of <2 W m^−1^ K^−1^,^64^ and MOF-74's thermal conductivity has been shown to vary with direction according to its pore structure.^67^ For MFM-300(M), the alignment of the cation chains parallel to the channels is likely to improve thermal conductivity along the channels, causing them to heat up and damage more quickly, resulting in the anisotropic degradation behaviour observed. The electrical conductivities of MOFs in turn have been shown to change with the adsorption of guest molecules, with a recent study showing that the adsorption of iodine by MFM-300(M) led to an increase in their electrical conductivities by up to 6 orders of magnitude due to the formation of conductive paths along the channels.^68^ However the choice of cation was crucial due to the need to tune suitable host-guest charge transfer.^69^ It would thus be interesting to see whether the incorporation of iodine into the channels of MFM-300(M) also translates to an increased electron beam stability.

Our work focuses on the stabilisation effect of different metal cations, but we recognise that different organic linkers will also have a strong effect on determining the MOF's electron beam stability. A recent study by Mücke *et al.* on planar Cu^2+^ MOFs with varying linker compositions confirmed that while replacing the hydrogen and nitrogen fragments in the linkers with oxygen and increasing the local density only had a small stabilisation effect, the substitution of oxygen with sulphur increased the stability of the resultant structure by a factor of 30.^37^ They surmised that the relative stabilities of these MOFs can be attributed to a balance between the bond strengths of the constituent elements of the linker, the caging effect from their packing densities, and their electrical conductivities. Though their study only considered Cu^2+^-containing MOFs, our results suggest that further improvements could potentially be made to the stability of these MOFs by using cations with lower solvent exchange rates.

At present, only a handful of studies have quantitatively evaluated the electron beam stability of MOFs,^37,70^ and no previous work has considered the effect of the metal cation species. The effects of coordination environment, crystal structure, and packing density have not yet been isolated and systematically investigated. Future degradation studies that explore these effects in isolation as well as the effect of metal cations in other MOF systems are required to build a deeper understanding of how all these various factors contribute to the electron beam stability of MOFs.

### Practical considerations for optimising TEM studies of MOFs

To promote successful future TEM imaging and diffraction studies of metal–organic frameworks, it is helpful to consider some of the practical considerations that need to be taken when storing, preparing, and characterising these MOF materials to improve the reliability of the data obtained. As we have shown that adsorbed solvents have a measurable effect on both the intensities and position of diffraction data, the prepared TEM grids should ideally be stored in a vacuum desiccator between experiments to reduce guest molecule adsorption.^71^ Before the samples are loaded into the TEM, it may also be beneficial to heat them to 100 °C (if thermal stability allows) or irradiate them using a UV lamp, ideally in a moderate vacuum, to encourage desorption of adsorbates.^55^ Additionally, leaving the sample overnight in the vacuum of the TEM before characterisation can be used to further encourage desorption of lingering guest molecules.^52^

As with any electron beam-sensitive specimen, calibration of the TEM electron dose and the use of the low-dose protocols, developed for cryo-EM imaging of proteins, is highly advisable. A Faraday cup should preferably be used to accurately measure the probe current used,^56^ with the phosphor screen current being an acceptable, albeit less accurate, alternative.^51^ Unlike single particle cryo-EM analysis, high-resolution imaging of MOF crystals requires manual alignment to a preferred zone axis, which can incur significant electron doses. Programs that can calculate the tilt needed to orient the sample correctly from an initial diffraction pattern are becoming increasingly available, and these will have great benefit for reducing unnecessary electron exposure.^29^ The usage of DED cameras,^31–34^ as well as techniques such as iDPC-STEM that can make more efficient use of the scattered electrons,^35,36^ are highly desirable to minimise the electron dose needed to achieve acceptable signal-to-noise ratios. It is important to note that preservation of the original structure of the MOF materials is vital for elucidation of structure–property relationships. Once the ligands collapse, the functionality of the MOF is largely compromised and therefore erroneous conclusions may be drawn from structural images even before the overall crystal structure is destroyed. All TEM analysis should therefore pay close attention to the potential for electron beam induced structural distortions, even at electron dose conditions below the critical dose.

Finally, the total electron fluence experienced by a region of interest is often missing from analysis of TEM imaging of MOFs. Wider reporting of this information, considering not only the acquisition time but the total irradiation time, starting from when the region is first exposed to the electron beam, would be highly valuable to drive forward progress in the field. Future work could study electron beam stability at different accelerating voltages as well as at cryogenic and elevated temperatures, to try to decouple beam-induced heating and other damage mechanisms.

## Conclusions

In conclusion, we have performed the first analysis of annealing and electron beam-induced structural degradation for the MFM-300(M) family of MOFs. We find that electron beam-induced structural changes are anisotropic, with the (nn̄0)-type lattice planes that run parallel to the one-dimensional pore channels being the most structurally stable feature. The thermal and electron beam stabilities for the Group 13 (Al, Ga, and In) MOFs are in good agreement, with MFM-300(Al) being the most stable and MFM-300(In) being the least. This behaviour is in line with the relative stability of their cation oxides, and agrees with previous work comparing the thermal stability of Cu-BDC materials.^58^ However, MFM-300(Cr) was shown to have an exceptionally high electron beam stability, twice that of MFM-300(Al), despite having a relatively lower thermal stability similar to that of MFM-300(Ga). The electron stability rankings of all four MOFs agrees well with the relative values for the inertness of the four metal cations when interacting with solvent, suggesting that electronic interactions between the cations and the electron beam as well as any generated radiolytic species are a greater contributor to degradation than beam-induced temperature changes. All four MOFs were found to undergo a gradual decrease in lattice parameter on electron beam irradiation, which was also reflected in the macroscopic size of the crystals. Excitingly, the lattice spacing contraction corresponding to the pore channel diameters in MFM-300(Cr) reached ≈10% before structural degradation of the pores, creating opportunities to tune the width of the MOF's one-dimensional channels around the chemically important size range of ≈1 nm.

It is interesting to note that our observations of electron beam-induced degradation correlate with the relative photocatalytic stabilities of the MFM-300(M) MOFs. Photocatalytic testing has shown that MFM-300(Cr) is the only member of this isostructural family to demonstrate an extended structural stability of up to 2 days under continuous photochemical exposure, whereas the Al, Ga, and In variants deteriorated rapidly under the same conditions.^72^ These results offer the intriguing possibility for electron beam-induced degradation studies to be applied in the future to understand and predict photocatalytic degradation.

Our results further suggest that the use of cations with high inertness may be a viable route to generating MOF structures with extended lifetimes for electrocatalytic and photocatalytic reactions. The knowledge gained in this work also provides valuable guidance on the effect of crystal thickness and MOF chemistry for improving our ability to investigate the local structure and chemistry of MOF materials with transmission electron microscopy, thereby supporting efforts to design new MOF structures and to tune their performance.

## Data availability

The data supporting this article have been included as part of the ESI.†

## Author contributions

G. C. and Y. C. carried out synthesis of the MOF samples. E.-P. T. was responsible for carrying out the experimental TEM work, with D.-T. N. providing training. N. C. drafted the initial Python script used for the analysis of the experimental data acquired, which E.-P. T. then developed and used for the data analysis. Y. C., J. H. C., S. P. T. and C. C. T. collected the VT-PXRD data. E. T. acquired the SEM cross-section images of the MOFs. C.A. acquired the STEM images of the MOFs. E.-P. T. was responsible for analysing all of the collected data. M. S., S. Y. and S. J. H. were responsible for the overall direction and design of the project. E.-P. T. and S. J. H. wrote the manuscript with contributions from all authors.

## Conflicts of interest

There are no conflicts to declare.

## Supplementary Material

TA-012-D4TA03302G-s001
