# Supplementary material for: Electron beam and thermal stabilities of MFM-300(M) metal–organic frameworks
Source: J Mater Chem A Mater. 2024 Jul 1;12(36):24165–74. doi: 10.1039/d4ta03302g (PMC11409654; doi:10.1039/d4ta03302g)
Supplement: TA-012-D4TA03302G-s001 [file TA-012-D4TA03302G-s001.pdf]

Electronic Supplementary Material (ESI) for Journal of Materials Chemistry A.
This journal is © The Royal Society of Chemistry 2024

Supplementary Information for
Electron Beam and Thermal Stabilities of MFM-300(M) Metal-Organic Frameworks

Eu-Pin Tien,[a,c] Guanhai Cao,[b] Yinlin Chen,[b] Nick Clark,[a] Evan Tillotson,[a] Duc-The Ngo,[a] Joseph H. Carter,[b] Stephen P. Thompson,[c] Chiu C. Tang,[c] Christopher S. Allen,[c,d] Sihai Yang,[b] Martin Schröder,[b] Sarah J. Haigh [a]*

Contents

SI 1: Sample Synthesis 2

MFM-300(Al) 2

MFM-300(Ga) 2

MFM-300(In) 2

MFM-300(Cr) 2

SI 2: Electron Flux Calibration for the Transmission Electron Microscope 3

SI 3: Method for Computational Quantitative Analysis of Diffraction Patterns 5

Electron Diffraction Analysis 5

VT-PXRD Analysis 6

SI 4: Further Electron Diffraction Pattern Analysis 7

Trends in Electron Beam Degradation 7

Peak Splitting in MFM-300(Cr) 8

SI 5: Crystal Cross-Section Analysis 10

SI 6: VT-PXRD Crystal Structure Analysis 12

Evolution of the Whole PXRD Pattern 12

Evolution of MFM-300 Lattice Spacings with Temperature 13

SI 7: Additional Supplementary Materials 17

Crystallographic Information Files 17

Electron Diffraction Pattern Series 17

References 18

a. *Department of Materials, The University of Manchester, Oxford Road, Manchester, M13 9PL, UK.*

b. *Department of Chemistry, The University of Manchester, Oxford Road, Manchester, M13 9PL, UK*

c. *Diamond Light Source Ltd, Diamond House, Harwell Science and Innovation Campus, Didcot, Oxfordshire OX11 0DE, UK*

d. *Department of Materials, University of Oxford, Oxford, OX1 3PH, UK*

* sarah.haigh@manchester.ac.uk

SI 1: Sample Synthesis

MFM-300(Al)

Biphenyl-3,3',5,5'-tetracarboxylic acid (H4BPTC; 0.264 g, 0.799 mmol), $AlCl_3 \cdot 6H_2O$ (0.528 g, 2.187 mmol), 10% HCl (3.3 ml), and dimethylformamide (DMF; 16.5 ml) were added to a 45 ml Parr bomb reactor.[1] The autoclave was sealed and heated to 145°C for 2 days, after which it was cooled for a day at room temperature. The resulting white microcrystalline powder was filtered *in vacuo*, then rinsed with DMF followed by acetone to give a white powder. Before conducting the TEM experiment, the powder was immersed in ethanol for 3 days for a thorough solvent exchange.

MFM-300(Ga)

H_4BPTC (21.8 mg, 0.067 mmol) and $Ga(NO_3)_3$ (38.0 mg, 0.148 mmol) were added to a 2:5:1 mixture of DMF, tetrahydrofuran (THF), and water (8 ml) acidified with 37% HCl (2 drops).[2] The mixture was then reacted in a pressure tube at 348 K for 72 hours. The white crystalline product was separated from the rest of the slurry by centrifugation and rinsed with acetone. Before conducting the TEM experiment, the powder was immersed in ethanol for 3 days for a thorough solvent exchange.

MFM-300(In)

H_4BPTC (330 mg, 1.00 mmol) and $In(NO_3)_3 \cdot 5H_2O$ (585 mg, 1.50 mmol) were mixed together in a DMF/MeCN (MeCN = acetonitrile) mixture (30 ml, 2:1 volume ratio) in a 250 ml glass pressure reactor.[3] The white slurry was acidified using concentrated HNO_3 (65%, 1.0 ml), and the vessel was then sealed and heated at 80°C for 48 hours. The resultant flaky white precipitate was washed with DMF and then acetone. Before conducting the TEM experiment, the powder was immersed in ethanol for 3 days for a thorough solvent exchange.

MFM-300(Cr)

$CrCl_3 \cdot 6H_2O$ (283 mg, 1.06 mmol) and H_4BPTC (70 mg, 0.212 mmol) were dissolved in water (10 ml), to which 12% HCl (1.5 ml) was added.[4] The reaction mixture was transferred to a 23 ml autoclave, sealed, and heated at 210°C for 72 h. The resultant blue powder was separated from the solution by filtration and rinsed with acetone. Before conducting the TEM experiment, the powder was immersed in ethanol for 3 days for a thorough solvent exchange.

SI 2: Electron Flux Calibration for the Transmission Electron Microscope

The electron diffraction experiments were performed on an FEI Tecnai G2 20 transmission electron microscope (TEM) equipped with a LaB_6 emission source and a Gatan Orius charge-coupled detector (CCD) camera calibrated to estimate electron flux. CCD cameras have a photomultiplier front layer that convert incoming electrons into photons, which are then detected by the CCD pixels as counts.[5,6] With an empty TEM sample holder in place, an aligned electron beam with a fixed diameter was set up, and the electron flux, J_{beam}, defined as the number of electrons passing through a unit area per unit time, was calculated under those beam settings using the following relationship:

$$J_{beam} = \frac{I_{beam}}{eA_{beam}}$$

Where:

- I_{beam} is the electron beam current, measured using the built-in phosphor screen,
- e is the elementary charge, $1.60217662 \times 10^{-19}$ C, and
- A_{beam} is the area illuminated by the beam.

With the CCD camera inserted, blank (no sample) images of the beam were acquired at magnifications where the beam covered the entirety of the camera's field of view. The area of the beam captured in the camera's field of view, A_{CCD}, could be determined from the magnification calibrations. Together with the exposure time t for the image, the total number of electrons N_e that contribute to the image is calculated as:

$$N_e = J_{beam}A_{CCD}t$$

By integrating the counts over all the pixels within the image, and varying the magnification and image acquisition times, a graph of the total number of electrons against the total number of CCD counts is plotted. By reference to this plot, the CCD count of a blank image can be used to determine the number of electrons contributing to that image, and therefore the electron flux the sample has been exposed to (**Figure S1)**. Acquiring blank (no sample) images before and after the image series acquisition from the MOF was used to guarantee the stability of the electron beam current during acquisition.

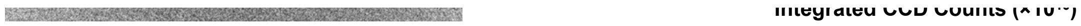

Figure S1: Calibration of CCD camera to estimate the electron flux used. a) Image of an aligned electron beam, acquired at a magnification where the entirety of the beam is in view. The beam is kept fixed at this size for the remainder of the calibration process. b) The diameter of the beam can be used to calculate the illuminated area. Together with the measured electron beam current, it is possible to estimate the electron flux of the beam. c) High-magnification image of the blank beam, used for measuring the count rate on the CCD camera. d) A plot of the total electrons contributing to the blank (no sample) image as a function of the total CCD counts for that same image. The linear trend demonstrates that the CCD camera is being operated safely below its saturation level, and CCD counts in vacuum can be used to estimate electron flux.

SI 3: Method for Computational Quantitative Analysis of Diffraction Patterns

Electron Diffraction Analysis

Each electron diffraction pattern data series was first loaded into the HyperSpy Python bundle [7] as an image stack. The image stack was median filtered using a 3 × 3 pixels mesh, then Gaussian filtered using a radius of 2 to reduce the impact of shot noise and dead pixels on the rest of the procedure. Values for the raw, median filtered, Gaussian filtered, and sequentially filtered image stacks were all stored in memory for subsequent processing.

Scikit-Image's peak finding algorithm [8] was applied to the first image to identify peaks and extract the coordinates at which they occurred. Peak tracking was then implemented by cropping a 64 × 64 pixels grid around the peak using the derived coordinates, then updating the coordinates for the current frame by applying the peak finding algorithm on the cropped image again. The maximum pixel value at this coordinate set was taken to be the amplitude of the peak for that frame for the processed and unprocessed data sets. This is illustrated in **Figure S2.**

The mean background for the cropped peak region was estimated by applying a circular mask of radius 16 pixels across the centre of the image, and taking the average intensity of the remaining pixels. The integrated intensity of the peak was then

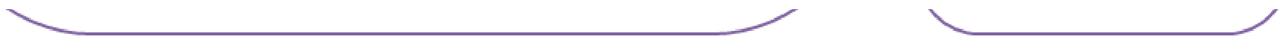

Figure S2: Peak fitting and tracking in an electron diffraction pattern. Left: Image of a diffraction pattern that has been subjected to the peak identification and tracking Python script, with the saved metadata for the experiment being displayed below. The algorithm is able to correctly identify the reflections present in the image in order of their intensities, but occasionally picks up noise. Right: Example of a reflection that is being tracked over the course of the electron beam degradation experiment, showing that the algorithm is able to effectively follow an assigned reflection even when its intensity has dropped to a low level.

determined by subtracting the mean background intensity multiplied by the number of pixels in the cropped image from the total intensity of the image. A 2D Gaussian model was also fitted to each peak, so that the calculated amplitude, mean background intensity, total integrated intensity, and fitting error could be recorded for each peak. This process was repeated for all frames in the image stack, with the diffraction peak being considered to have disappeared if:

a) the peak finding algorithm fails to return coordinates, and
b) the shift in coordinates between two consecutive slices is too great (typically >10 pixels between frames; this is indicative that the algorithm is now detecting noise or background fluctuations).

The intensity measurements acquired were then saved in an Excel spreadsheet and plotted using Matplotlib.[9]

The HyperSpy Python script used in this work was able to detect most of the peaks present in the image, but occasionally picks up noise rather than true diffraction, the data of which was removed manually. As the crystals were at different orientations, different sets of reflections were obscured by the beam stop. In order to enable more accurate cross-comparison, only the reflections common to all the data sets were compared, with the obscured reflections and their diametric opposites being omitted. The remaining reflections in each frame were then summed together to yield the total intensity for that frame.

VT-PXRD Analysis

Analysis of the VT-PXRD data was also carried out using Python following a similar routine to the electron diffraction pattern analysis. The VT-PXRD data was first imported as a NumPy array [10] as a series of 2θ values and their corresponding intensities. The x-ray wavelength used was 0.826459(2) Å, with a zero difference of -0.00745°, and this was used to convert the 2θ values into the reciprocal lattice spacings they represented via Braggs' Law.

We focused on the $1\ 1\ 0$, $1\ 1\ 2$, and $0\ 0\ 4$ reflections due to their relation to the $2\ 2\ 0$, $1\ 1\ 2$, and $0\ 0\ 2$ reflections studied in the electron diffraction experiments, with $1\ 1\ 0$ in particular being an indicator of the a- and b-axes unit cell parameter (which determines the one-dimensional channels pore diameter) and $0\ 0\ 4$ indicating the length of the unit cell along the c- axis (hence the length of the channels). The $0\ 0\ 2$ reflection was not present in the PXRD patterns due to it being kinematically forbidden, but was present in the electron diffraction pattern likely due to dynamic electron scattering.[11] Using published unit cell data of the MFM-300 MOFs studied,[1–4] the reciprocal lattice spacings corresponding to the peaks of interest were calculated, and the arrays were cropped around these spacings.

The scikit-image peak finding algorithm was again applied to this cropped region, and the position of the peak, its amplitude, its integrated intensity, and the local background were measured and tracked across the temperature series, until the signal could no longer be discerned from the local background noise. This data was then saved as an Excel spreadsheet for further analysis.

SI 4: Further Electron Diffraction Pattern Analysis

Trends in Electron Beam Degradation

Using the data array acquired via analysis of the electron diffraction pattern series, it was possible to plot and observe the trends of a wide variety of features within the diffraction patterns. Due to the vastly different rates at which the reflections decayed, we integrated the reflections common to all the cross-compared MOFs to see how the overall diffraction patterns decayed as a function of the total electron fluence.

Figure S3a shows the combined intensity data for the representative Al, Ga, In, and Cr MOFs plotted on a linear-linear scale. It is evident that the diffraction intensities undergo exponential decay in intensity with increasing electron exposure as displayed on the log-log plot in **Figure S3b** and log-linear plot in **Figure S3d**. The log-linear plot shows that the intensity decay can be approximated to an exponential function, with the best fit seen at the later stages of degradation. The decay profile upon initial electron exposure is less consistent, likely a result of the crystals tilting during the degradation process.

Figure S3: Plots of the total integrated intensity of the studied MOFs as a function of total electron fluence using a variety of scales: a) linear-linear, b) log-log, c) normalised linear, and d) log-linear. Legend in (a) applies to all plots.

Metal Cation	Critical Fluence for Each Reflection ($e^{-}\cdot Å^{-2}$)			Critical Fluence of Integrated Pattern ($e^{-}\cdot Å^{-2}$)
	$\sigma_c(0$	$\sigma_c(1$	$\sigma_c(2$	
**Al**	134	160	413	330
**Ga**	88	74	159	189
**In**	61	61	143	147
**Cr**	160	145	910	1111

Table S1: The critical electron fluences at which the intensities of the , , and reflections drop to 10% of their maximum intensities in the MFM-300(M) crystals.

The normalised-linear plot (**Figure S3c**) was most useful for measuring the 10% intensity threshold that we have used as the critical electron fluence for the integrated diffraction pattern. The values of the critical fluence for the individual reflections discussed in the main paper as well as that of the integrated pattern are shown here in **Table S1**. As can be seen, the critical fluence for the individual reflections span a wide range, so the critical fluence of the integrated diffraction pattern serves as a more robust measure of the overall decay of the diffraction pattern.

Peak Splitting in MFM-300(Cr)

Minor peak splitting along the $n\,\bar{n}\,0$ directions could be observed in the diffraction pattern (see **Figure S4**). The distance between the outer pair of peaks correspond to a $(1\ 1\ 0)$ lattice spacing of 10.60 Å, which matches well with the values derived from published unit cell data of MFM-300(Cr),[4] whereas the inner pair correspond to a $(1\ 1\ 0)$ lattice spacing of 10.75 Å (see **Table S2**). This peak splitting suggests differences in the pore diameter along the one-dimensional channels of MFM-300(Cr), which have been reported for the other isostructural members of this family recently.[12]

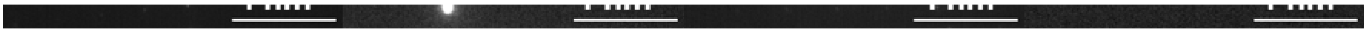

Figure S4: Peak splitting observed in the diffraction patterns of MFM-300(Cr). a) and b) TEM images of two pristine MFM-300(Cr) crystals, which were imaged down the zone axis. i) Selected area electron diffraction patterns of the crystals, which have been aligned to correspond to the crystal orientation in TEM imaging. ii) Close-up image of the areas highlighted in i) (red squares), showing the slight splitting of peaks (red circles) along the reciprocal lattice directions.

Sample	Set of Peaks	Width (nm^{-1})	Number of Spacings Averaged	Reciprocal Lattice Spacing (nm^{-1})	Lattice Spacing (Å)
**1**	Inner	9.309	10	0.931	10.743
	Outer	9.432	10	0.943	10.602
**2**	Inner	9.300	10	0.930	10.752
	Outer	9.431	10	0.943	10.604
**3**	Inner	9.295	10	0.930	10.758
	Outer	9.430	10	0.943	10.605

Table S2: Measurements of the difference in spacing between the inner and outer split peaks observed in the set of reflections. The outermost set of reflections match the that of pristine MFM-300(Cr), which have a lattice spacing of 10.60 Å,[4] whereas the inner ones correspond to an expanded spacing of ≈10.75 Å.

SI 5: Crystal Cross-Section Analysis

Focused ion beam scanning electron microscopy (FIB-SEM) characterisation of the cross-sectional geometry of an MFM-300(Cr) crystal was conducted via an FEI Helios 660 FIB-SEM. MOF crystals were deposited on an oxidised silicon substrate. A suitable crystal was selected using SEM imaging (**Figure S5a**) and coated with an electron beam-assisted deposited (EBAD) Pt strap followed by an ion beam-assisted deposited (IBAD) strap of Pt to minimise damage done to the MOF during ion beam thinning. Additionally, the deposition of Pt inhibits curtaining, facilitating flat cross-sectioning which is necessary for accurate cross-sectional measurements.

Cross-sectional thinning of the MOF started using an 800 pA ion beam current with an accelerating voltage of 30 kV for the milling of bulk trenches. Then, 400 pA at 30 kV was used to thin the crystal to within 200 nm of the target cross-section region. Finally, 80 pA at 30 kV was used to complete the milling process, with a 0.5° under-/over-tilt further ensuring a flat cross-section. SEM imaging was conducted at 5 kV with a 1.6 nA probe current, a 4 mm working distance, and a dwell time per pixel of 300 ns.

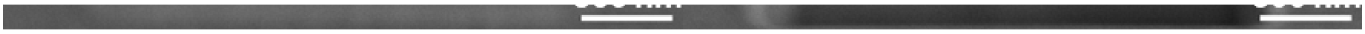

Figure S5: FIB-milling of MFM-300(Cr) crystals. a) Low-magnification SEM images showing the distribution of MFM-300(Cr) of various sizes, acquired at a tilt angle of 52°. b) Schematic diagram showing the viewing geometry within the FIB-SEM and the relationship between the projected apparent height observed (   ) and the crystal's actual height (   ). c) Uncorrected image of a FIB-milled MOF cross-section, viewed at a tilt angle of 52°.A coating of Pt was applied via EBAD followed by IBAD in order to prevent curtaining during the milling process and ensure a flat cross-section. d) Tilt-corrected image of c), which creates a virtual parallel projection of the cross-section to allow for accurate measurements to be taken. The square cross-section of the MFM-300(Cr) crystal is demonstrated with equal dimensions along the   and   directions.

Cross-sectional images of the MFM-300(Cr) crystals were then acquired with and without the activation of the cross-section tilt correction algorithm, which adjusts the image to compensate for the specimen tilt to ensure a virtual, parallel projection of the cross-section as well as properly calibrated image measurements (see **Figure S5b**). From the tilt-corrected image in **Figure S5d**, the square cross-section of the MFM-300(Cr) crystal can be clearly seen, justifying the assumptions made for the thickness analysis of the crystals during the electron beam degradation studies. The height and width of 10 crystals were measured digitally using FIJI, and are presented along with their calculated aspect ratios in **Table S3** below, showing that the crystals have square cross-sections with only a small margin of error caused by poor definition of the crystal edges in the

Specimen	Width (nm)	Height (nm)	Aspect Ratio (Width Height)
**1**	347.5	348.1	1.00
**2**	440.2	443.4	0.99
**3**	1010.8	1034.7	0.98
**4**	439.2	431.8	1.02
**5**	500.0	482.6	1.04
**6**	433.1	417.4	1.04
**7**	477.7	447.1	1.07
**8**	393.8	410.3	0.96
**9**	283.8	310.4	0.91
**10**	432.4	454.0	0.95

Table S3: Cross-section measurements of 10 MFM-300(Cr) crystals acquired using the workflow shown earlier. The aspect ratios of these crystals are close to 1, with a small margin of error, showing that the growth rates of the crystals along the and axes are virtually identical.

SEM images due to the image contrast.

SI 6: VT-PXRD Crystal Structure Analysis

Evolution of the Whole PXRD Pattern

High-resolution variable temperature PXRD (VT-PXRD) of the four MOFs was conducted at the I11 beamline of the Diamond Light Source using position-sensitive detectors (PSD) and monochromated radiation (λ = 0.826459(2) Å). The acetone-exchanged MOF samples were loaded into capillary tubes of 0.7 mm diameter, which were treated at 150°C and 10^{-10} bar for 12 hours. VT-PXRD data of the MOFs were then collected *in vacuo* using a heating rate of 300°C·hr^{-1} from 25°C to 800°C. One minute was given for the temperature of the MOF to stabilise after each heating process.

The VT-PXRD spectra for the Al, Ga, In, and Cr MOFs at different temperatures are shown in **Figure S6**. From the spectra, it can be seen that the specimens are pure, with the exception of MFM-300(Cr), which shows unexpected peaks at low reciprocal lattice spacings. These have been attributed to a zeolite that may have been present as a contaminant in the

Figure S6: VT-PXRD data for the Al (a), Ga (b), In (c), and Cr (d) MFM-300 MOFs, with the reflections analysed in detail for this study (bold) and other intense peaks visible in the spectra labelled below the Al figure. MFM-300(In) was found to have decomposed into cubic In_2O_3 at around 500°C (c), as shown by the peaks at 2.42 nm^{-1} and 3.42 nm^{-1} (¤), which correspond to the and reflections of In_2O_3. Additional diffuse peaks (*) could be seen in the MFM-300(Cr) data that persisted up until 400°C before fading. These peaks have been attributed to contamination when loading the MFM-300(Cr) into the container used for the characterisation.

diffractometer. MFM-300(In) was the only MOF that formed any crystalline phase during thermal degradation, decomposing into cubic In_2O_3 above 500°C (**Figure S6c**), with the reflections found to correspond to the $2\ 1\ 1$ and $2\ 2\ 2$ reflections.[13]

Evolution of MFM-300 Lattice Spacings with Temperature

Figure S7 shows graphs plotted via Matplotlib of the VT-PXRD reflections to be cross-compared with those in the electron diffraction patterns. The peaks were observed to sharpen and increase in intensity with increasing temperature before decreasing at higher temperatures, indicating that the MFM-300 crystals are somewhat disordered at room temperature, but become more crystalline upon heating up until they thermally degrade. The reciprocal lattice spacings associated with those reflections are shown to change with increasing temperature.

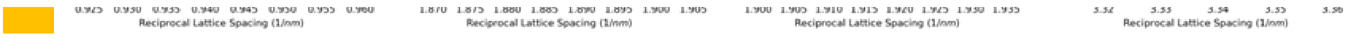

Figure S7: Analysis of the VT-PXRD background-subtracted diffraction intensities as a function of temperature for the MFM-300(M) MOFs. The peak of the reflections can be seen to shift with temperature, indicating a change in the lattice spacings associated with those temperatures. The reflections also become sharper with increasing temperature up until a certain threshold, indicating reduced crystalline disorder with heating. However, beyond that threshold, thermal degradation occurs in earnest, and they begin to lose their crystallinity, as indicated by the fading of the reflections.

Figure S8 shows the evolution of these peak intensities in further detail, where the real and normalised intensity values, along with the decay rate of the normalised intensities, are plotted together. Plotting the normalised intensity values shows that all the reflections for a given MOF degrade isotropically, decaying to the same fraction of the maximum registered value at the same temperature. The normalised intensity decay rate was found to be a robust method of defining the critical temperature, with similar critical temperatures defined for each MOF from all four individual reflection plots.

Figure S8: Evolution of the , , , and peak intensities as a function of the temperature. Left: The real integrated intensities of the reflections. Middle: The integrated intensities, normalised to their peak values. The critical threshold for degradation here is defined as the temperature when the intensity drops to 10% of its maximum value (labelled). Right: The decay rate of the normalised intensity, with the peak decay rates labelled. The decay rates of the MFM-300(Cr) reflections are particularly noisy, persisting over an extended temperature range with no clear peak value.

Figure S9 shows the plots of the lattice spacings and unit cell volumes of the four studied MOFs. The reciprocal lattice spacings for the $n\ n\ 0$ set of reflections correspond to the diagonals along the a- and b-axes of the unit cell, i.e., the pore cross-section, and increase with temperature, indicating a shrinkage of the pore channels. The $1\ 1\ 2$ and $0\ 0\ 4$ lattice spacings, on the other hand, were shown to have expanded slightly, as shown by the decreasing reciprocal lattice spacing, indicating expansion along the c-axis. This is conclusive evidence that the MFM-300 MOFs exhibit anisotropic thermal expansion behaviour, thought to be attributed to desorption of solvent species.

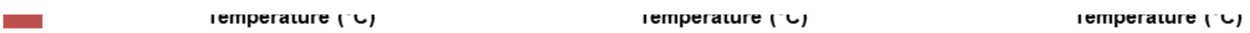

Figure S9: Plots of the change in lattice spacings (, ,) and the unit cell volume of the MOFs as a function of temperature. Left: Actual values of the lattice spacings and unit cell volumes. Middle: The evolution of said values as a percentage change of the initial value. Right: The rate of change of the percentage change.

Having observed the lattice spacing changes along the a-, b-, and c-axes of the unit cell, we can determine the overall change in unit cell volume of the MOFs during heating. MOFs are known to exhibit negative thermal expansion (NTE) due to the metastable nature of their porous structures.[14] As the MFM-300 MOFs are known to possess a tetragonal unit cell structure ($a = b \neq c$), the measured lattice spacing values for the $1\ 1\ 0$ and $0\ 0\ 4$ reflections (d_{110} and d_{004}) were used to calculate their unit cell volumes (V_{cell}):

$$V_{cell} = d_{001} \times {d_{100}}^2 = 4d_{004} \times \left(\frac{d_{110}}{\sin(45°)}\right)^2$$

Equation S1: Volume of the MFM-300 unit cell calculated from the lattice spacings measured for the $1\ 1\ 0$ and $0\ 0\ 4$ reflections based on a tetragonal unit cell.

The volumes calculated using the experimental data acquired match well with those reported in literature (see **Table S4**). Despite the noisy data, the Ga and Cr MOFs show a slight overall expansion, whereas the Al and In MOFs experience a slight shrinkage, with all changes being on the order of <0.5%. The first order differential plots shown are quite noisy, but do not suggest any discrete high-temperature phases.

Metal Cation	(Å)	(Å)	(Å³)	Molecular Weight
**Al**	14.817(10)	11.817(8)	2594.35	414.19
**Ga**	15.0174(7)	11.9111(11)	2686.22	499.67
**In**	15.4886(8)	12.3439(13)	2961.26	589.86
**Cr**	14.99047(3)	11.99050(3)	2694.44	464.22

Table S4: Lattice parameters of the MFM-300 unit cells for Al,[1] Ga,[2] In,[3] and Cr,[4] determined by X-ray diffraction. All four materials have isostructural tetragonal crystal lattices with the space group.

SI 7: Additional Supplementary Materials

A brief summary of the additional files uploaded as part of this journal article submission can be found here.

CIF File	Description
**MFM-300(Al) CCDC1856083.cif**	Unit cell structure of vacant MFM-300(Al) (formerly also known under the designation of NOTT-300(Al)).<br>CCDC Link: https://www.ccdc.cam.ac.uk/structures/Search?Ccdcid=CCDC1856083
**MFM-300(Cr) CCDC1952277.cif**	Unit cell structure of vacant MFM-300(Cr).<br>CCDC Link: https://www.ccdc.cam.ac.uk/structures/Search?Ccdcid=CCDC1952277
**MFM-300(Ga) CCDC951539.cif**	Unit cell structure of vacant MFM-300(Ga).<br>CCDC Link: https://www.ccdc.cam.ac.uk/structures/Search?Ccdcid=CCDC951539
**MFM-300(In) CCDC1475893.cif**	Unit cell structure of vacant MFM-300(In).<br>CCDC Link: https://www.ccdc.cam.ac.uk/structures/Search?Ccdcid=CCDC1475893

Table S5: CIF files used for indexing the electron diffraction patterns acquired, and to generate simulated XRD patterns to compare the VT-PXRD data against. A link to their respective entries on the Cambridge Crystallographic Data Centre (CCDC) is provided.

Crystallographic Information Files

File Name	Description
**MFM-300(Al) 2019-03-26 T20 A2 TEM_003_E7_0006 8-bit.tif**	Representative diffraction pattern series of MFM-300(Al).<br>First image taken at 6 min after initial illumination.
**MFM-300(Cr) 2019-09-30 T20 A1 TEM_003_N1_0012 8-bit.tif**	Representative diffraction pattern series of MFM-300(Cr).<br>First image taken at 12 min after initial illumination.
**MFM-300(Ga) 2019-05-13 T20 A1 TEM_003_G3_0007 8-bit.tif**	Representative diffraction pattern series of MFM-300(Ga).<br>First image taken at 7 min after initial illumination.
**MFM-300(In) 2018-05-24 T20 A2 TEM 003 D6 0005 8-bit.tif**	Representative diffraction pattern series of MFM-300(In).<br>First image taken at 5 min after initial illumination.

Table S6: Compressed 8-bit TIFF image stacks of the electron diffraction series used for cross-comparison of the 4 MOFs have also been provided. Images were acquired on an FEI Tecnai G2 20 equipped with a LaB_6 filament and operating at 200 kV. Images were acquired at 1 min intervals, using an exposure time of 30 s at a constant electron flux of 0.1 $e^-/Å^2s$.

References

1 H. G. W. Godfrey, I. da Silva, L. Briggs, J. H. Carter, C. G. Morris, M. Savage, T. L. Easun, P. Manuel, C. A. Murray, C. C. Tang, M. D. Frogley, G. Cinque, S. Yang and M. Schröder, *Angew. Chemie Int. Ed.*, 2018, **57**, 14778–14781.

2 C. P. Krap, R. Newby, A. Dhakshinamoorthy, H. García, I. Cebula, T. L. Easun, M. Savage, J. E. Eyley, S. Gao, A. J. Blake, W. Lewis, P. H. Beton, M. R. Warren, D. R. Allan, M. D. Frogley, C. C. Tang, G. Cinque, S. Yang and M. Schröder, *Inorg. Chem.*, 2016, **55**, 1076–1088.

3 M. Savage, Y. Cheng, T. L. Easun, J. E. Eyley, S. P. Argent, M. R. Warren, W. Lewis, C. Murray, C. C. Tang, M. D. Frogley, G. Cinque, J. Sun, S. Rudić, R. T. Murden, M. J. Benham, A. N. Fitch, A. J. Blake, A. J. Ramirez-Cuesta, S. Yang and M. Schröder, *Adv. Mater.*, 2016, **28**, 8705–8711.

4 L. Briggs, R. Newby, X. Han, C. G. Morris, M. Savage, C. P. Krap, T. L. Easun, M. D. Frogley, G. Cinque, C. A. Murray, C. C. Tang, J. Sun, S. Yang and M. Schröder, *J. Mater. Chem. A*, 2021, **9**, 7190–7197.

5 A. R. Faruqi and G. McMullan, *Q. Rev. Biophys.*, 2011, **44**, 357–390.

6 D. B. Williams and C. B. Carter, in *Transmission Electron Microscopy: A Textbook for Materials Science*, Springer US, Boston, MA, 2009, pp. 115–126.

7 F. de la Peña, E. Prestat, V. T. Fauske, P. Burdet, J. Lähnemann, P. Jokubauskas, T. Furnival, M. Nord, T. Ostasevicius, K. E. MacArthur, D. N. Johnstone, M. Sarahan, J. Taillon, T. Aarholt, pquinn-dls, V. Migunov, A. Eljarrat, J. Caron, C. Francis, T. Nemoto, T. Poon, S. Mazzucco, actions-user, N. Tappy, N. Cautaerts, S. Somnath, T. Slater, M. Walls, F. Winkler and H. W. Ånes, 2022.

8 S. van der Walt, J. L. Schönberger, J. Nunez-Iglesias, F. Boulogne, J. D. Warner, N. Yager, E. Gouillart and T. Yu, *PeerJ*, 2014, **2**, e453.

9 J. D. Hunter, *Comput. Sci. Eng.*, 2007, **9**, 90–95.

10 C. R. Harris, K. J. Millman, S. J. van der Walt, R. Gommers, P. Virtanen, D. Cournapeau, E. Wieser, J. Taylor, S. Berg, N. J. Smith, R. Kern, M. Picus, S. Hoyer, M. H. van Kerkwijk, M. Brett, A. Haldane, J. F. del Río, M. Wiebe, P. Peterson, P. Gérard-Marchant, K. Sheppard, T. Reddy, W. Weckesser, H. Abbasi, C. Gohlke and T. E. Oliphant, *Nature*, 2020, **585**, 357–362.

11 D. B. Williams and C. B. Carter, in *Transmission Electron Microscopy: A Textbook for Materials Science*, Springer US, Boston, MA, 2009, pp. 221–233.

12 X. Li, J. Wang, N. Bai, X. Zhang, X. Han, I. da Silva, C. G. Morris, S. Xu, D. M. Wilary, Y. Sun, Y. Cheng, C. A. Murray, C. C. Tang, M. D. Frogley, G. Cinque, T. Lowe, H. Zhang, A. J. Ramirez-Cuesta, K. M. Thomas, L. W. Bolton, S. Yang and M. Schröder, *Nat. Commun.*, 2020, **11**, 4280.

13 M. Marezio, *Acta Crystallogr.*, 1966, **20**, 723–728.

14 C. S. Coates and A. L. Goodwin, *Mater. Horizons*, 2019, **6**, 211–218.
